# High-resolution rural poverty mapping in Pakistan with ensemble deep learning

**DOI:** 10.1371/journal.pone.0283938

**Published:** 2023-04-04

**Authors:** Felix S. K. Agyemang, Rashid Memon, Levi John Wolf, Sean Fox

**Affiliations:** 1 Department of Planning and Environmental Management, University of Manchester, Manchester, United Kingdom; 2 Social and Economic Survey Research Institute, University of Qatar, Doha, Qatar; 3 School of Geographical Science, University of Bristol, Bristol, United Kingdom; University of Maryland at College Park, UNITED STATES

## Abstract

High resolution poverty mapping supports evidence-based policy and research, yet about half of all countries lack the survey data needed to generate useful poverty maps. To overcome this challenge, new non-traditional data sources and deep learning techniques are increasingly used to create small-area estimates of poverty in low- and middle-income countries (LMICs). Convolutional Neural Networks (CNN) trained on satellite imagery are emerging as one of the most popular and effective approaches. However, the spatial resolution of poverty estimates has remained relatively coarse, particularly in rural areas. To address this problem, we use a transfer learning approach to train three CNN models and use them in an ensemble to predict chronic poverty at 1 km^2^ scale in rural Sindh, Pakistan. The models are trained with spatially noisy georeferenced household survey containing poverty scores for 1.67 million anonymized households in Sindh Province and publicly available inputs, including daytime and nighttime satellite imagery and accessibility data. Results from both hold-out and k-fold validation exercises show that the ensemble provides the most reliable spatial predictions in both arid and non-arid regions, outperforming previous studies in key accuracy metrics. A third validation exercise, which involved ground-truthing of predictions from the ensemble model with original survey data of 7000 households further confirms the relative accuracy of the ensemble model predictions. This inexpensive and scalable approach could be used to improve poverty targeting in Pakistan and other low- and middle-income countries.

## Introduction

The impact of Covid-19 on lives and livelihoods has accelerated social protection support efforts by governments and non-governmental organisations across the globe. High resolution poverty mapping supports these efforts [1]. However, an alarming half of all countries do not have access to sufficient data produce such maps [2]. Census data has been the traditional source of for generating spatial estimates of deprivation. Yet censuses are expensive and infrequent [3, 4], which undermines their utility in rapidly developing countries experiencing rapid socioeconomic and demographic change. Sampled surveys, such as Demographic and Health Surveys (DHS) and the World Bank’s Living Standards Measurement Surveys (LSMS), have emerged as a popular alternative source for generating nationally and sub-nationally representative socio-economic and health data (see for example [1, 5, 6]). However, these surveys have relatively small samples, and spatial coverage is limited. Significantly expanding the coverage of such data is costly and challenging for most low- and middle-income countries [7].

To address these challenges researchers have explored alternative and less costly approaches to estimating economic activity at subnational levels. Early efforts used luminosity from nighttime lights (NTL) as a proxy for measuring economic activity [8–11]. luminosity has been found to be correlated with wage income [12] and asset wealth [13] at various spatial scales. However, nighttime lights are highly limited in observing variations in economic activity and living standards in LMICs [5, 12, 14].

More recently, improvements in machine learning and computer vision have led to efforts to use daytime satellite imagery to generate spatial estimates of wealth and deprivation [15]. Many of these approaches make use of deep learning techniques such as Convolutional Neural Networks (CNN). For example, [1] used CNN to map wealth across 20,000 villages in Africa; [16] used CNN with boosted regression trees to predict asset wealth in LMICs; [5] combined CNN with ridge regression to measure consumption expenditure in five African countries; [17] used the approach to estimate GDP in US counties; and [18] mapped poverty in Uganda with a combination of CNN and logistic regression. This approach is particularly valuable in data-scarce regions, mainly in LMICs (see [6, 16, 19–21]). A key benefit of the approach is that models can be trained with publicly available satellite imagery as inputs, including Landsat [22], Google Static Maps [5], DMSP and VIIRS [16], making them not only scalable but also inexpensive to apply in the real world.

Many of these CNN models were used to generate estimates in both urban and rural areas. Yet there are strong *a priori* reasons to expect substantial differences in the types of visual information required to accurately predict variation in economic activity or living standards between and within rural and urban areas. For example, technologies of production vary widely between agricultural and non-agricultural contexts, as do indicators of consumption (such as dwelling size). While broad variations in economic activity or welfare between urban and rural areas may be visible from the sky, it is more difficult to observe differences *within* either context from above. Urban areas tend to be more socio-economically heterogeneous, with building features as well as morphology generally reflecting socio-economic characteristics of households [23–25]. Rural areas exhibit less architectural and morphological variation, although may have greater variation in landscapes that contain information on household living conditions. It is therefore unlikely that a single model applied to satellite imagery could reflect intra-urban *and* intra-urban variation in household welfare at a high spatial resolution. Put differently, it is not terribly difficult to make broadly accurate spatial estimates of relative living standards between urban and rural areas with satellite imagery given their distinct economic characteristics. By contrast, it is challenging to generate accurate spatial estimates at a high resolution *within* each of these contexts.

There has been some progress in this area, notably the mapping of asset wealth across 20,000 villages in Africa [1]. However, the spatial resolution of [1]’s work, as with other studies using CNN, is coarse—especially in rural contexts (see [6, 17, 18]). The resolution of Yeh et al. (2020) CNN model is 6.72km × 6.72km. Even though the ‘micro-estimates’ of wealth from [16] are presented at 2.4km × 2.4km resolution, the underlying DHS data, the target layer or label for the CNN model, was aggregated to 4.8km × 4.8km grid cells in urban areas and 9.6km × 9.6km grid cells in rural areas. The coarseness of the existing deep learning models is largely influenced by the sources of the economic data used for training the networks. Data from Demographic and Health Surveys, the dominant source, is spatially distorted up to 2km in urban areas and 5km in rural areas to preserve the anonymity of households. Similarly, the geographical coordinates of the World Bank’s Living Standard Measurement Study (LSMS) contain up to 5km noise. Existing models therefore resort to coarser spatial resolutions to reduce their sensitivity to this small-scale locational noise.

Policy makers in LMICs seeking to target livelihood interventions in rural areas at a much finer scale will have major challenges relying on existing measurements of economic well-being. Pakistan is one such case. Pakistan’s Sindh Province, home to an estimated 48 million people, has established a Strategic Social Protection Unit (SPSU) and assigned it resources to develop a targeting strategy. The SPSU has also been tasked with identifying eligible households in rural Sindh for cash relief in response to shocks, such as Covid-19 and monsoon floods.

Building on existing efforts, we develop and train three CNN models and combine them in an ensemble CNN to generate small-area estimates (1km^2^) of chronic poverty in rural Sindh to support such targeting. We utilize an extensive georeferenced household survey containing data on assets and poverty scores for 1.9 million anonymized households in Sindh province. Asset based poverty and wealth indices are generally seen as less noisy and more stable, especially in the long term, than those based on consumption [26, 27]. Whilst the survey is comprehensive in coverage in the districts where it was executed, it was conducted in just half of the districts in Sindh and has significant spatial distortions making our task comparable to past studies that used noisy datasets like the DHS.

Our results demonstrate that an ensemble transfer learning approach can be used to predict chronic poverty across arid and non-arid rural regions with promising results at 1km^2^ resolution—a much higher resolution than that achieved by previous studies. Our inexpensive and scalable approach could be used to improve high resolution poverty targeting in Pakistan and other low- and middle-income countries.

## Defining and measuring rural poverty in Pakistan

While definition and measurement of poverty remains contested [28], we use a poverty measure based on household assets, which is both conceptually robust and practical. This approach builds on the ‘basic needs’ concept that constitutes the primary framework for defining national poverty lines [29]. Historically, the use of a basic needs approach to poverty measurement was limited to rich countries. From the 1980s, the institutionalization of the Living Standards Measurement Surveys (LSMS), promoted by the World Bank, regularly provided the data necessary for poverty line measurement in developing countries as well [29]. However, these surveys are financially and technically demanding and provide data at very course resolution. As Covid-19 recently demonstrated, many policy makers in developing countries require high resolution measures of poverty that can be collected quickly, accurately and cost effectively.

“Quick and dirty” measures of poverty [30], have therefore developed alongside the “long and clean” measures based on large household surveys. Participatory Poverty Assessments, for example, became very popular among NGOs after the 1970s. Based on the principles of ‘optimal ignorance’ (importance of knowing what is not worth knowing) and proportionate accuracy (much survey data has a degree of accuracy that is unnecessary), these measures provided a shortcut, avoiding more expensive direct and time-consuming investigations. Since then, participatory poverty assessments have been conducted in many countries in East and South Asia, Africa and Latin America (See [31–33]).

This kind of data can then be used to improve targeting by providing information on relative need through a ‘proxy means test’ (PMT) to predict whether a household is poor (i.e. in need of government support) or not. This approach is valuable in LMICs with limited household data [34]. The World Bank, in particular, uses detailed household surveys (e.g. LSMS) to establish PMT models for individual countries [35], which can then be used to produce household-level estimates of poverty with ‘quick and dirty’ data collected at higher frequency and lower cost.

One of the most popular rapid data collection methodologies is the Simple Poverty Score (SPS) developed by [36] with support from the Ford Foundation and Grameen Foundation, which has now been used in 63 countries [37]. The SPS is similar in approach to the USAID’s Poverty Assessment Tool in method but claims a greater degree of transparency and ease of use [38]. It uses a short survey and weights estimated from nationally representative surveys using logistic regression.

The SPS requires information in three main areas: the location of a household, household member characteristics, and household assets such as air conditioners, refrigerators, vehicles, agricultural land and livestock. Data on these characteristics is then compressed into twelve indicators. For example, ownership of refrigerators, freezers and washing machines is lumped into one binary indicator, which takes a value of 1 if a household possesses any of the three assets. Similarly, air conditioners and heaters are compressed into one indicator. Each indicator is then assigned a weight. The exact choice of the twelve indicators and the weights assigned to them depend on the context. Logistic regression models using data from Living Standards Measurement Surveys are typically used to identify which 12 indicators are the best predictors of poverty in any given country and period (see [36] for details). The coefficients from the regression are then transformed into weights for each indicator. The total score (the sum of individual scores) can then be related to the probability that a household is poor by using a simple statistical table. A local pro-poor organization can then implement a small household survey based on just these 12 indicators, calculate a poverty score, and determine eligibility for a household to receive subsidized goods and services.

The SPS has received much attention from World Bank Programming as the Bank’s twin goals of eliminating extreme poverty and inequality require measuring poverty rates in specific populations targeted by development programs worldwide. Considering the time and cost required for using poverty measures based on large scale survey data, and the data and technical demands of using small area estimations, the SPS has become the post popular solution for project specific poverty estimation [37].

The development of the SPS for Pakistan using PSLM 2005/06 is documented in [39], and an update using the PSLM 2007/08 is documented in [40]. Each household receives a score between 0 and 100, with higher scores indicating lower levels of deprivation. In January 2009, The Government of Pakistan adopted the poverty SPS as the targeting tool for the Benazir Income Support Program, the flagship cash transfer program. The cut-off score for poverty was decided as 17.5, at which 16.3 percent of families–about 5.9 million–would be covered. This cutoff was chosen to align with an estimated national poverty headcount of 17% at the time [40]. In general, the rural poverty rate in Pakistan has always exceeded the national poverty rate by about 5 to 6 percentage points [41].

The SPS on which this paper is based was collected from 1.9 million households in 14 districts in Pakistan’s Sindh province, as part of the Sindh Union Council Economic Strengthening Support (SUCESS) Programme. The SUCCESS Programme covered eight out of the province’s 24 districts; data were collected in an additional six were by the Government of Sindh (GoS) and Sind Rural Support Organization (SRSO) under the People’s Poverty Reduction Program (PPRP). Table 1 lists the enumerated districts and presents the household count from the Population Census 2017, which was conducted soon after the poverty scoring.

**Table 1:** Simple Poverty Score coverage in districts.

Districts	Rural HH (Census 2017)	SPS Enumeration Coverage
Badin	282909	75.1
Jamshoro	104518	74.8
Dadu	216911	78.5
Matiari	109997	70.9
Sujawal	136805	73.0
Tando AY	113185	73.0
Tando MK	104297	69.9
Thatta	150588	88.7
Kamber	155566	94.4
Larkana	142358	85.0
Mirpur Khas	205234	75.6
Umerkot	164990	77.6
Sanghar	267383	77.3
Khairpur	279258	95.7

Source: SUCCESS/PPRP and authors’ calculations using Population Census 2017

As can be seen in Table 1, the SPS survey coverage is very high but not complete. The data were collected through computer-assisted personal interviews (CAPI) at the doorstep of each household using Android Tablets. Household information was collected from a household member older than 18 years, with preference for the head or the spouse of the head of the household. GPS readings were taken at the end of each interview.

**Fig 1:**
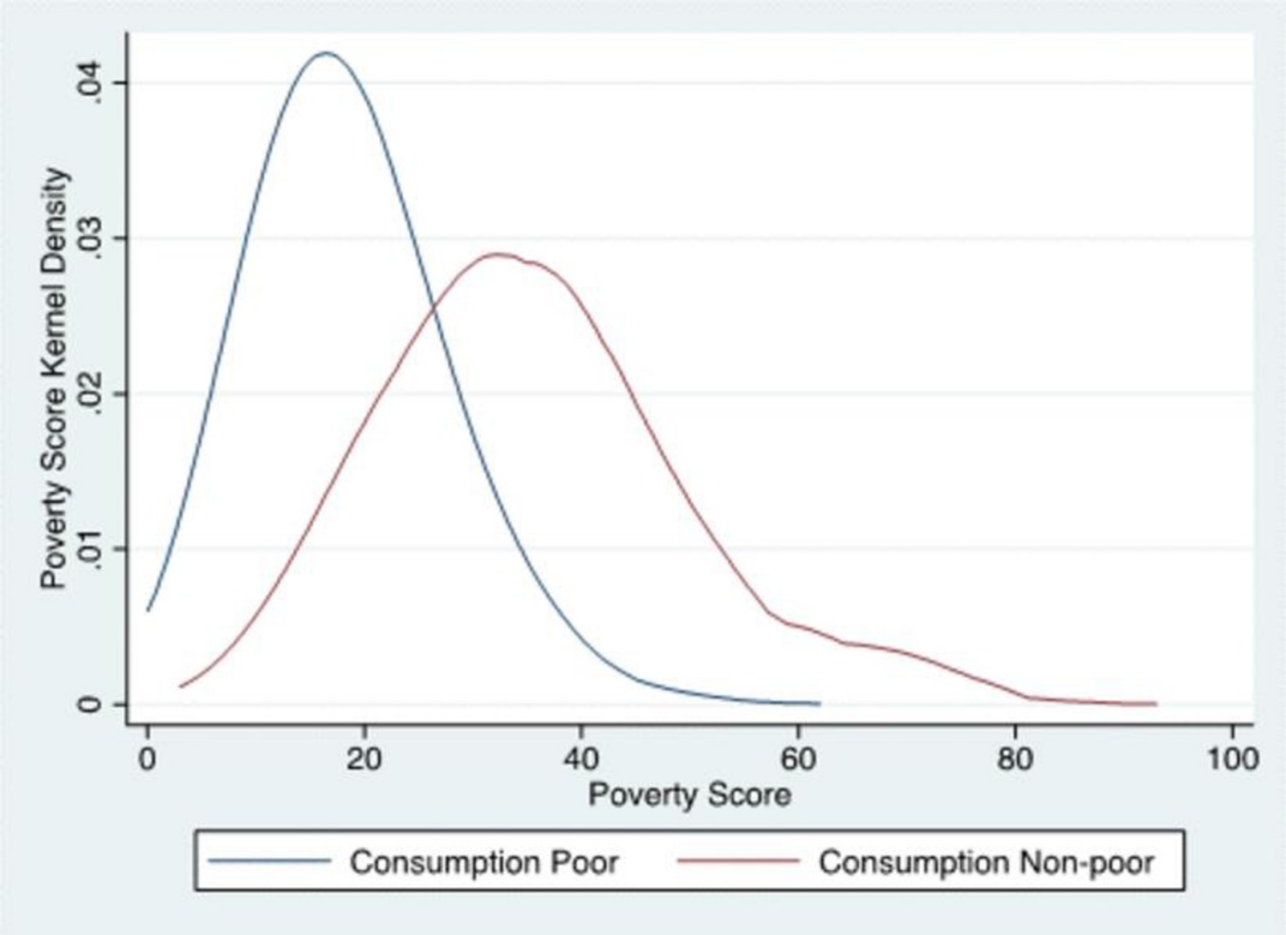
The Simple Poverty Score and consumption poverty. Source: Authors’ estimations using HIES 2015–16.

We can evaluate the relationship between household SPS scores in our sample with the consumption-based measure used for Pakistan’s official poverty line [42] using data from the Household Integrated Economic Survey (HIES) from 2015–16. Fig 1 shows the distribution of the poverty score for the consumption poor and consumption non-poor using the government definition. There is overlap between poverty scores of the poor and non-poor, which means there is a risk of inclusion errors at low poverty scores (i.e., counting the non-poor as poor due to a low SPS). Above a SPS of approximately 62 the probability of being poor drops to zero, but the probability of being non-poor is ‘reasonably’ high above a poverty score of approximately 30.

Given the different dimensions of life measured in the SPS, it is natural to explore relationship between the SPS and a measure of multidimensional poverty, which has gained prominence in subnational poverty research and policy in recent years. The SPS explicitly uses poverty on non-monetary dimensions to predict consumption / basic-needs poverty. Multidimensional poverty, on the other hand explicitly recognizes that poverty along social dimensions *may not necessarily* accompany reductions in consumption poverty. Vision 2025, which institutionalized a multidimensional poverty index (MPI) to informs policymaking in Pakistan, was designed with the explicit aim of balancing progress on monetary measures of poverty with that in the social dimension [41]. For example, a household is considered deprived in education if a child is not going to school because schools are far away or are unaffordable. Similarly, a household is deprived if health facilities are too far away or lack enough staff to serve new clients.

Although the MPI was supposed to be updated every two years, consistent with the frequency of the district-level representative PSLM survey, the only measure of MPI currently available is based on the 2014–15 survey. Households deprived on 33% of the weighted indicators are categorized as poor. Table 2 presents the poverty headcounts based on MPI and SPS for each district for which SPS data is available. As the table shows, the level of poverty, or the headcount, is generally much lower with the SPS than it is with the MPI. Nevertheless, a correlation coefficient of 0.74 indicates a strong linear relationship between the two measures of poverty.

**Table 2:** District level poverty headcounts based on MPI (2014–15) and Simple Poverty Score (2015/16/17).

District	MPI Headcount	SPS Headcount	Median SPS	Mean SPS
Larkana	42.0	28.8	25.0	25.9
Dadu	51.4	40.9	21.0	22.5
Khairpur	51.6	28.7	24.0	25.9
Jamshoro	55.6	32.5	23.0	24.8
Matiari	62.1	28.6	25.0	26.2
Sanghar	66.8	34.9	23.0	24.0
Tando AY	67.3	31.3	23.0	25.3
Mirpurkhas	68.9	39.7	21.0	22.5
Kambar Shahdadkot	72.0	41.0	21.0	22.3
Badin	74.8	40.7	21.0	21.8
Tando MK	78.4	38.0	21.0	23.1
Thatta	78.5	40.1	21.0	22.2
Sujawal	82.0	46.9	19.0	20.6
Umerkot	84.7	44.9	19.0	20.9

Source: The MPI Headcount is from [42]. SPS Headcounts are based on authors’ calculations using SUCCESS/PPRP data.

## Methodology for estimating rural poverty with transfer deep learning

### Ethics statement

"The study conducted a survey involving households. The data was, however, aggregated into 1 square kilometer cells making it impossible to identify specific households. The study received written ethics approval from the Institutional Review Board, Lahore University of Management Sciences.

Approval number: LUMS-IRB/05/30/2022/RM-FWA-00019408"

### Consent statement

We first sought the verbal consent of households prior to their participation in the survey. The following statement was read to all households. *“*[Greetings!] *my name is*……. *I am from Gallup Pakistan*. *We are conducting a survey to understand people’s livings standards [lit*. *how people live] in collaboration with a Lahore based University*. *This interview will take about 15–20 minutes*. *The information you provide will not be shared with anyone and will only be used for academic purposes*. *Do you agree to participate in this survey*?*”* Only households who answered yes to the question participated in the survey.

### Target layer / labels data

The Social Protection Strategic Unit of Pakistan’s Sindh Province has a goal of identifying households in rural Sindh eligible for cash relief, the kind of support where households severely affected by Covid-19 and Monsoon floods are given financial assistance. Following these, we perform a binary classification task that predicts whether a household in a given 1km^2^ grid cell is likely to be chronically poor using the median poverty score of the cell. Thus, each 1km^2^ cell represents a single “training scene;” a model will learn from input about the scene from different data sources and predict the poverty status of the median household in that scene. For the poverty status variable, the PPRP report suggests the following categorization: 0–11 Extremely poor/destitute, 12–18 Chronically poor, 19–23 Transitory poor, 24–100 Non poor. We binarized these classes, categorising a cell with 0–18 PSC score as “chronically poor,” and those with 19 or greater as “not chronically poor.” Thus, we predict whether the median poverty score of a cell is below 19 (chronically poor) or 19 and above (not chronically poor). In machine learning classification tasks, there is often a decision to be made between optimizing for recall or precision. For a poverty classifier with good recall, most of the areas that are *truly* “chronically poor” would be predicted as chronically poor. In contrast, a classifier with good precision would instead focus on making sure that most of the predicted “chronically poor” areas are actually “chronically poor.” The SPSU’s cash transfer program, as with many poverty interventions, seeks to ensure that everyone who needs support gets support, which drove us to optimize recall accuracy over precision in cases of trade-off between the two.

We accessed the SPSU data through the office of the Chief Minister’s Coordinator for Social Protection. While the data were meant to represent exclusively rural households, visual inspection revealed that some surveyed areas were *de facto* urban in nature, usually peri-urban settlements on the edge of medium-sized cities. Consultation with local experts in Pakistan confirmed that these areas were surveyed because their administrative status was ‘rural’ at the time. In line with our objective to map poverty in rural areas, we dropped these *de facto* urban observations. We also dropped all observations falling within or intersecting the boundaries of an “urban centre” as defined by the Global Human Settlement Layer (GHSL). Specifically, we used data from the 2019 Settlement Model (SMOD) of the GHSL to extract the urban centre layer [43]. We identified and dropped 95,271 observations falling within this urban centre layer. We also dropped 183,656 observations with (1) poverty scores exactly equal to zero–understood to be errors–or (2) GPS locational accuracy error over 20 meters, and left out 5,531 surveys conducted before 2016. Our final sample contains 1.67 million individual georeferenced observations.

Two types of spatial errors became evident while cleaning the data: (1) spatially diffuse GPS coordinates for individual settlements (often in fields or on roads), suggesting that the coordinates were not captured at the actual location of the household/settlement, and (2) unrealistically dense concentrations of observations in towns and cities from enumeration areas, suggesting that enumerators may have congregated at a location to upload data and accidentally assigned that location to all surveys that had been collected that day.

To create a target layer for the CNN model, we computed the median PSC score for observations falling within each 1km^2^ grid cell. Given the spatial errors described above, we opted for a spatial resolution of 1km^2^, substantially finer than the resolution used in the studies discussed earlier, but large enough to reduce the influence of spatial noise. This left 35,730 cells across Sindh with PSC observations. To preserve the anonymity of households, we eliminated 3,120 cells with fewer than 3 observations. We also dropped 329 cells with 300 or more observations, which probably reflected new urban settlements. Table 3 shows the descriptive statistics of median poverty scores for ~ 1.67 million households and 1km^2^ grid cells.

**Table 3:** Descriptive statistics of PSC observations and target layer (1km^2^ grid cells).

	Min	Max	Mean	Std	1^st^ Quartile	Median	3rd Quartile
Household count (1km^2^ grid cells)	3	299	47	48	14	31	62
Median poverty score (1km^2^ grid cells)	3.5	80	21	5.5	18	21	24
Poverty scores (not gridded)	1	100	23	11	15	22	30

### Input data

We used three openly accessible inputs: daytime satellite imagery, nighttime satellite imagery, and accessibility data. Previous studies have shown that daytime satellite imagery and NTL offer important information about the economic geography of areas [5, 6, 10]. For data on daytime satellite imagery, we accessed 10m × 10m resolution Sentinel 2 images from ESA’s Copernicus Open Access Hub via QGIS. The images were captured between January and April 2016, contemporaneous with the SPS data collection period. All tiles except 1 had less than 1 percent cloud cover. The tiles were processed into true colour images and mosaicked into a single raster. In addition to RGB bands, Sentinel has Near-Infrared (NIR) and Short-wave Infrared (SWIR) bands containing additional information that could facilitate learning and predictions. However, including these two bands would constrain the usage of pre-trained weights and make it extremely challenging to use a transfer learning approach. For NTL data, we used the 2016 median VIIRS Annual VNL V2 product from the Earth Observation Group [44]. The original resolution of the VIIRS image was ~ 500m × 500m, so we resampled it down to 10m × 10m to match the Sentinel 2 resolution. Finally, we used accessibility layer reflecting travel time to settlements with 5,000–10,000 population, which was extracted from global accessibility map [45].

### Transfer learning with convolutional neural networks

To predict whether the median household in each cell is chronically poor we employed a transfer learning approach involving four CNN models: (1) ResNet-50 (2) ResNet-50V2 (3) ResNet-101, and (4) an ensemble model comprising the first three. Past studies have used transfer learning techniques to map poverty and economic wellbeing with good results [5, 6, 18]. These studies employ a two-step approach where an existing CNN model is first used to predict nighttime light intensity using daytime satellite imagery as input. In the process, the CNN learns to extract predictive features from the daytime satellite imagery which are subsequently used as inputs in a regression to predict the final target label. We followed the approach of [1] by training the CNN models end-to-end using the three inputs (daytime and nighttime satellite and accessibility).

The three ResNet architectures were chosen for their high performance on the ImageNet image classification challenge, with its ensemble model topping several competitions in 2015, including ImageNet Large Scale Visual Recognition Challenge, ImageNet Detection, and ImageNet Localisation. Besides, ResNet architecture has been used by previous studies to predict economic wellbeing with a high accuracy [1]. Complex image classification tasks such as predicting poverty at 1km^2^ resolution with a spatially noisy dataset normally require more layers of neural networks. However, as network layers increase, many models experience degradation, with accuracy stagnating and declining. By utilising “skip connections” ResNet architecture provides an avenue for adding more layers without experiencing much decline in prediction quality. ResNet50 uses a bottleneck design that makes it more computationally efficient than earlier versions of ResNet such as ResNet34. We also selected ResNet50V2 as it performed better than ResNet50 in the ImageNet classification challenge. We added ResNet101 to have more layers in the range of models we chose.

We predicted the poverty status for each cell using each of the three individual models and the ensemble model in turns. For the ensemble model, we used the modal predicted status of a cell. All the models have three inputs (Sentinel 2 images, nightlights, and accessibility), and the data within each cell has100 x 100 pixels. The models were initialized with weights trained on ImageNet.

For a given model and input, we extracted features with the corresponding ResNet architecture, performed global average pooling to reduce the extracted features, and added dense layers for classification. We then concatenated the final layers of each trained input and added a fully connected layer, which outputs a binary classification for each cell: chronically poor or not chronically poor. To minimize overfitting, we introduced dropouts prior to the final layer that randomly eliminated neurons.

The models were trained using ADAM to optimize the overall accuracy. A batch size of 16 and learning rate of 0.00005 were used in the training for all the models. The models were trained over 30 epochs with an early stopping mechanism that allows the models to stop after 10 continuous epochs if there is no improvement in the validation accuracy. The weights from the best performing epochs were retained. We implemented and trained the models using the Keras and Tensorflow libraries in Python.

The data and codes used in this paper can be accessed here on Figshare.

## Results and discussion

We adopted a three-stage validation framework. First, we used each model to generate predictions for random holdout test samples and compared their performance across a number of accuracy metrics, including recall, precision, overall accuracy, ROC curves, and Area Under Curve (AUC). Second, we used a spatial cross-validation approach: whole districts were omitted from the training of each model and then used for out-of-sample testing. As with the first approach, we compare the cross-validation performance of the models across a range of accuracy metrics. Last, we used the model with the best performance from the first two validation approaches to generate predictions for Ghotki, a district with no PSC data, conducted an original survey involving 7194 households sampled from 174 1km^2^ grid cells, and compared our predictions with the survey data.

**Fig 2:**
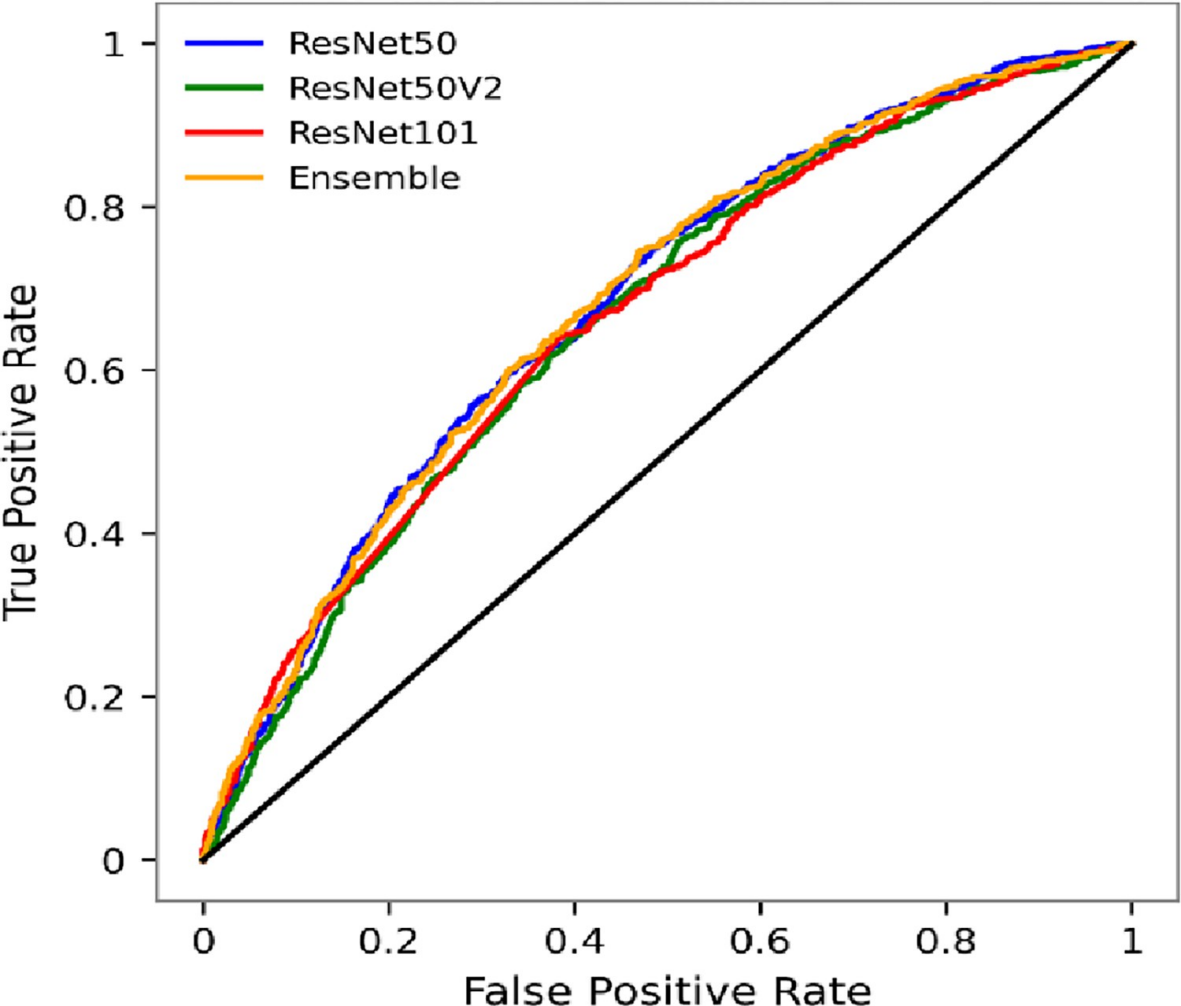
Comparison of CNN models ROC curves.

For the first stage, 30 percent of the cells across Sindh had PSC scores < 19, and 70 percent ≥ 19. To avoid bias in the training datasets, we sampled ~ 9600 cells (the count of cells with PSC < 19) from the latter so we have the same number of samples for each class. The dataset was split into 15,596 training, 1,732 validation, and 1,925 test samples. The performance of each model is summarized in key metrics presented in Table 4 and Fig 2.

**Table 4:** Comparison of CNN models.

	ResNet50	ResNet50V2	ResNet101	Ensemble
Recall	0.76	0.69	0.65	0.71
Precision	0.61	0.61	0.62	0.62
Accuracy	0.62	0.61	0.62	0.63
AUC	0.68	0.66	0.67	0.69

ResNet50 performs best in minimizing exclusion errors in identifying the chronic poor, with recall accuracy of 76 percent, followed by the Ensemble model (71 percent). ResNet101 and the Ensemble perform a percentage point better than ResNet50 and ResNet50V2 in the precision metric. Similarly, the Ensemble model has the highest overall accuracy and AUC, but only a percentage better than ResNet50 in both metrics. Thus, the performance of the Ensemble and ResNet50 are close as reflected in the ROC curves in Fig 2.

For the second stage of validation, we randomly split the datasets into six folds (denoted “kf”s), with each fold containing two districts. We then trained each model on five folds (ten randomly-selected districts) and tested the models’ out-of-sample accuracy for one fold (two districts). The folds were rotated for each iteration such that all districts were used for both training and testing at the end of sixth round. This approach paints a better picture of the likely performance of the model when used to generate predictions in districts with no poverty data. We also compare the performance of the models at each cross-validation iteration with random predictions. We produced random predictions for the test sample, repeated 1000 times for each iteration, and the median score for recall, precision and overall accuracy were computed.

Table 5 shows the performance of each model and random predictions for each iteration. ResNet50V2 and the Ensemble model produced high and stable results in minimizing exclusion errors (ie. maximising recall) across the iterations. ResNet50V2 recorded recall accuracy of 70+ in five out of six folds, followed by the Ensemble model with recall accuracy of 70+ in four out of six folds. Despite performing well with the Ensemble in the first validation stage, ResNet50’s recall accuracy performance is less stable in completely out-of-sample validation as shown in Table 5. Indeed, at iteration kf4, ResNet50’s recall is lower than that of a lottery (random predictions). In minimizing inclusion errors, the Ensemble model, ResNet50 and ResNet101 generated the most consistent results. ResNet50V2’s precision accuracy is not as stable as its recall. For instance, it performed three points lower than random predictions at iteration 6 (kf6). The Ensemble model, ResNet50 and ResNet101 generated consistent and moderately high results in overall accuracy across all the iterations. As with precision metric, the performance of ResNet50V2 in overall accuracy fluctuated, dropping below random predictions for iteration 6 (kf6).

**Table 5:** Cross-validation comparison of CNN models and random predictions for the chronically poor.

	Recall (%)					Precision (%)					Overall Accuracy (%)					AUC			
	ResNet50	ResNet50V2	ResNet101	Ensemble	Random	ResNet50	ResNet50V2	ResNet101	Ensemble	Random	ResNet50	ResNet50V2	ResNet101	Ensemble	Random	ResNet50	ResNet50V2	ResNet101	Ensemble
kf1	66	80	64	72	50	41	40	41	41	34	55	52	56	55	50	0.57	0.60	0.59	0.62
kf2	70	77	78	80	50	44	41	40	43	34	60	55	54	58	50	0.67	0.64	0.65	0.68
kf3	82	72	70	81	50	30	31	30	32	33	51	56	55	54	50	0.69	0.65	0.67	0.70
kf4	49	67	57	59	50	43	41	41	42	34	59	54	56	58	50	0.61	0.59	0.57	0.62
kf5	75	71	67	75	50	37	35	35	37	33	56	53	55	56	50	0.61	0.63	0.61	0.64
kf6	58	80	55	65	50	34	27	33	33	30	66	49	66	64	50	0.65	0.67	0.62	0.69

The performance of the models for recall, precision and overall accuracy shows the Ensemble model as the most stable performer across all the iterations. This is also shown by the AUC metric, where the Ensemble consistently performs above 0.6, with a range of 0.62 to 0.7. The Ensemble has the highest median AUC (0.66) and is the only model out of the four to record up to 0.7 AUC. ResNet50 and ResNet50V2 have AUC lower than 0.6 at kf1 and kf4 respectively, and ResNet101 has similar AUC at both iterations. Thus, the cross-validation does not only show the Ensemble as the best performing model across the metrics, but also the most reliable. We therefore selected the Ensemble model as the best performer out-of-sample and used it to predict chronic poverty in a district with no poverty data, the third validation stage.

**Fig 3:**
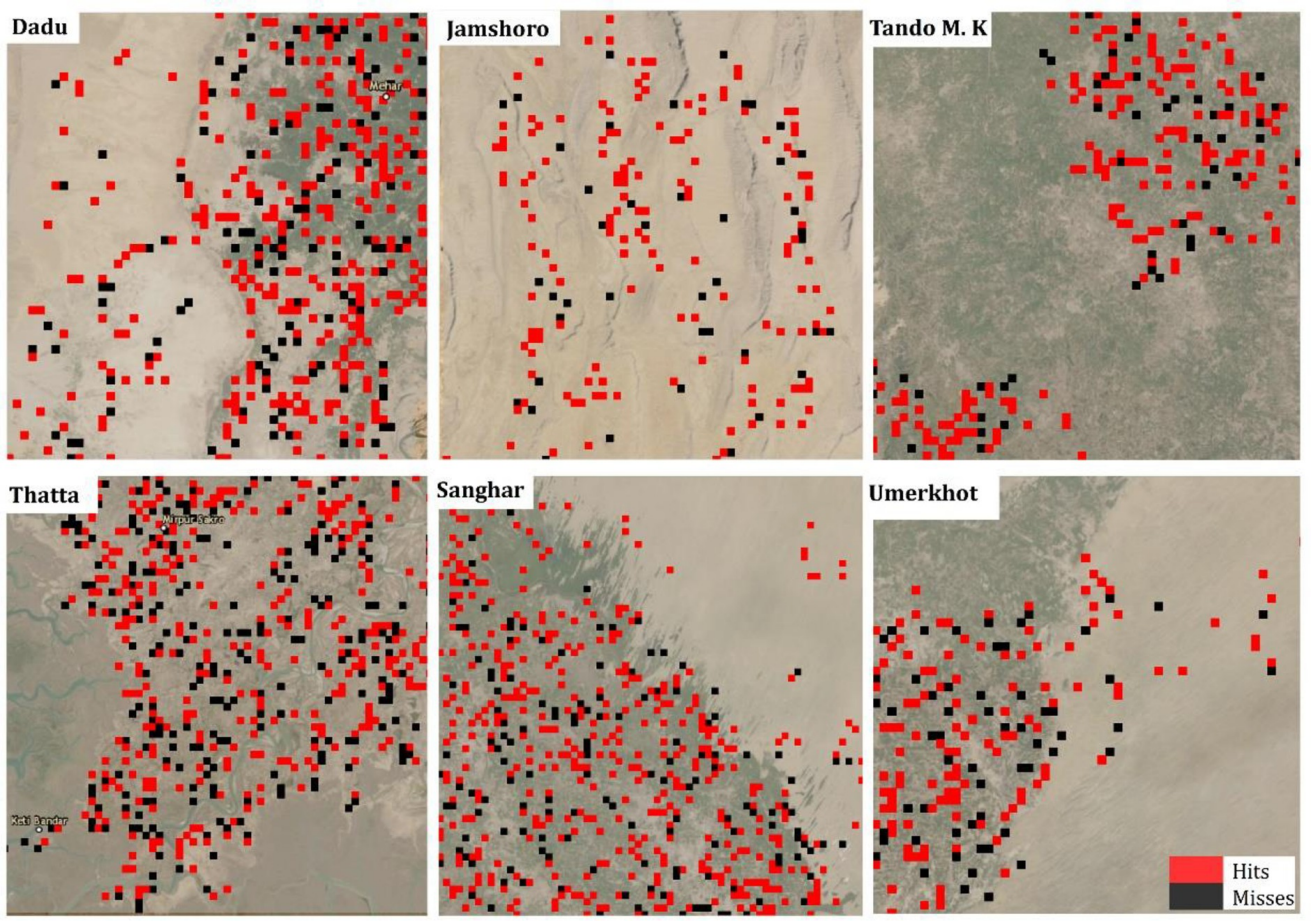
Cross-validation performance of ensemble CNN model in selected districts. Hits: observed poor and predicted poor. Misses: observed poor and predicted not poor.

Prior to the third validation, we mapped the Ensemble model’s cross-validation recall results to assess its performance in minimizing exclusion errors in both arid and non-arid regions. The model’s recall performance is high in both arid and non-arid ecological regions of the province as shown in Fig 3. The highest recall (81 percent) is observed in kf3, which used predominantly non-arid districts (Tando M. K and Tando A. Y) forout-of-sample testing. The second highest recall (80 percent), however, is found in kf2 with test districts comprising of Matiari and the arid Jamshoro. The model’s recall is also high in test districts that have both arid and non-arid zones. For instance, the model recorded 75 percent recall accuracy in Sanghar and Mirphurkhas (Kf6), test districts with both ecological zones. Thus, the ensemble CNN model produces generally good results across different ecological contexts, which is important because it shows the model is not biased against a particular ecological zone.

**Fig 4:**
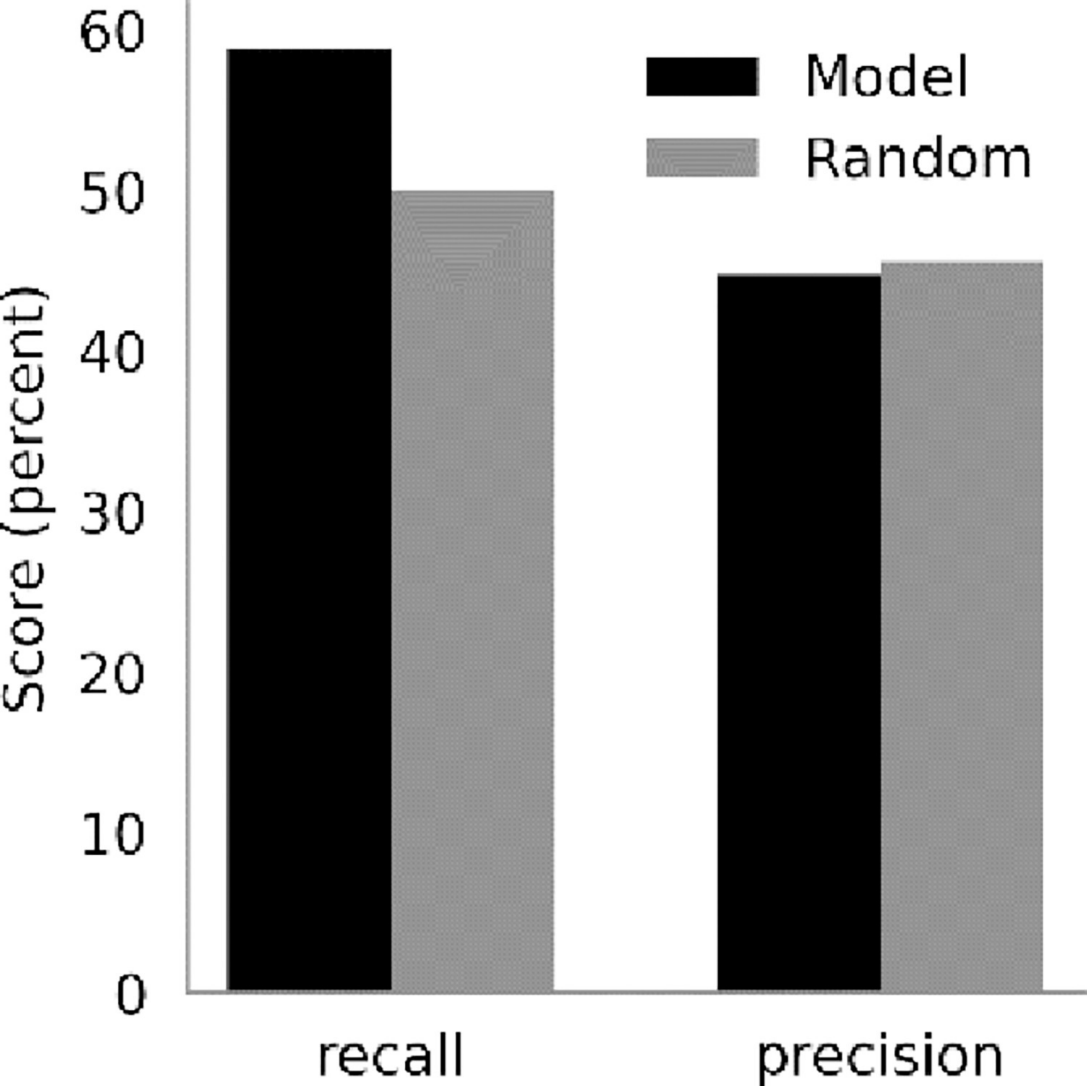
Ground truthing results of CNN model versus random predictions.

Validation of existing CNN applications are mainly limited to cross-validation approaches like those described above. We go a step further to conduct a true out-of-sample validation exercise using an original survey conducted by Gallup Pakistan in Ghotki, a district with no PSC data with both arid and non-arid ecological conditions. Prior to the survey, we used the ensemble model to generate predictions for all habitable 1km^2^ grid cells of Ghotki and randomly selected approximately 200 cells for enumeration. Using the PSC methodology, we constructed poverty scores from the original survey data and used it as a benchmark to assess the performance of the model. After data cleaning, the final ground-truth survey validation set comprised approximately 7000 household from 174 cells. We also executed thousands of random predictions and compared it with the CNN model as shown in Fig 4.

The ensemble model’s performance in the key recall metric is 59 percent while that of the random predictions (median) is 50 percent. The two are virtually at par for precision metric: 44 percent for the model and 45 percent for the random predictions. The fact that the ensemble model performed nine points better than random prediction in the recall metric is significant considering that the model was trained on 2016–2019 PSC data while the ground-truthing survey was conducted in 2022. Moreover, unlike the original survey used for the validation, the PSC data used to train the model contain substantial spatial errors as described earlier.

The results across all three validation stages compare favorably with previous research in the field. The most relevant comparison is [18], which adopts a classification (rather than regression) approach to poverty prediction with transfer learning. Their model identifies the poor in Uganda with 66 percent recall accuracy at 39 percent precision. The results from holdout test samples randomly drawn across Sindh indicates that our ensemble model performs considerably better: five points higher in recall, and 23 points higher in precision. Thus, the model outperforms [18] in minimizing both inclusion and exclusion errors. This is even more significant considering the spatial resolution of our model is 10 times finer than that of [18], which is 10km × 10km. Moreover, [18]’s poverty mapping includes urban and rural areas, making it less challenging than differentiating economic characteristics within rural areas as done here.

The model’s performance from the rigorous cross-validation is even better in recall accuracy in four of the six iterations. The model’s recall is at least 14 points higher for two of the iterations (Kf2 and Kf3), and six points higher or more for another two (Kf1 and Kf5). The recall of the model is lower than [18]’s for only one iteration. The model’s precision is higher for three iterations (Kf1, Kf2 and Kf4) and lower in the other three. A potential reason for the ensemble model’s comparatively average precision performance is inclusion errors in the underlying SPS. As shown in Fig 1, poverty scores below 19 often reflect households that are both asset-poor and consumption-poor, but occasionally some asset-poor households are not consumption-poor. Our target measure (the poverty scorecard) is chiefly asset-based, making it extremely challenging to identify these consumption-poor households when training on asset-poor scores from the sky. However, given that our primary concern is recall, this mediocre performance on precision is not a significant concern.

## Conclusion

To overcome the limitations of traditional census and survey-based approaches to poverty mapping, and to contribute to the growing body of work that seeks to exploit advancements in deep learning and remote sensing, we developed an ensemble CNN to predict chronic poverty in rural Pakistan. In doing so, we sought to address two key limitations of previous studies by (a) focusing on differentiating between chronically poor not chronically poor areas within rural areas, and (2) doing so at a much higher spatial resolution than previous studies.

We used transfer learning to develop and compare the performance of three CNN models, including ResNet50, ResNet50V2, ResNet101, and an ensemble model comprising the three individual models to map chronic poverty at 1km^2^ resolution in rural Sindh, Pakistan. The models were trained on data from Pakistan’s comprehensive but spatially noisy and geographically incomplete Simple Poverty Scorecard, with satellite imagery (daytime and night-time) and accessibility data as inputs. We have demonstrated that the CNN model trained on publicly available inputs can generate good prediction of poverty at a much finer scale in rural areas, even when the target data is noisy, and that an ensemble model offers the most stable performance. Rigorous validation, including a fully out-of-sample validation stage involving ground truthing of predictions with an original survey, show that the ensemble model performs well in minimizing exclusion errors across both arid and non-arid regions, which are important in determining livelihood and lifestyle patterns in rural Pakistan. Altogether, our low cost and scalable approach to predicting rural poverty can improve how social welfare interventions are targeted in data scare LMICs. This approach could be further improved by the collection of data with less spatial noise for training transfer learning models, which are likely to become increasingly popular in such contexts to inform and evaluate social welfare interventions.
